# Mother and son cases of Bickerstaff’s brainstem encephalitis and fisher syndrome with serum anti-GQ1b IgG antibodies: a case report

**DOI:** 10.1186/s12883-021-02159-y

**Published:** 2021-03-20

**Authors:** Hirokazu Natsui, Makoto Takahashi, Kentaro Nanatsue, Sakiko Itaya, Keisuke Abe, Akira Inaba, Satoshi Orimo

**Affiliations:** Department of Neurology, Kanto Central Hospital, 6-25-1 Kami-Yoga, Setagaya-ku, Tokyo, 158-8531 Japan

**Keywords:** Bickerstaff’s brainstem encephalitis, Fisher syndrome, Familial, Anti-GQ1b antibody, *Haemophilus influenzae*, Case report

## Abstract

**Background:**

Bickerstaff’s brainstem encephalitis (BBE) and Fisher syndrome (FS) are immune-mediated diseases associated with anti-ganglioside antibodies, specifically the anti-GQ1b IgG antibody. These two diseases potentially lie on a continuous spectrum with Guillain-Barré Syndrome (GBS). There are some reports of family cases of GBS and fewer of FS. However, there are no reports of family cases of BBE and FS.

**Case presentation:**

We report a familial case of an 18-year-old son who had BBE and his 52-year-old mother diagnosed with FS within 10 days. The son showed impaired consciousness 1 week after presenting with upper respiratory symptoms and was brought to our hospital by his mother. He showed decreased tendon reflexes, limb ataxia, albuminocytologic dissociation in his spinal fluid, and positive serum anti-GQ1b antibodies. *Haemophilus influenzae* was cultured from his sputum. He was diagnosed with BBE and treated with intravenous immunoglobulin (IVIg) therapy, which led to an improvement in symptoms. The mother presented with upper respiratory symptoms 3 days after her son was hospitalized. Seven days later, she was admitted to the hospital with diplopia due to limited abduction of the left eye. She showed mild ataxia and decreased tendon reflexes. Her blood was positive for anti-GQ1b antibodies. She was diagnosed with FS and treated with IVIg, which also led to symptomatic improvement.

**Conclusions:**

There are no previous reports of familial cases of BBE and FS; therefore, this valuable case may contribute to the elucidation of the relationship between genetic predisposition and the pathogenesis of BBE and FS.

## Background

Bickerstaff’s brainstem encephalitis (BBE) is an immune-mediated disease of the brainstem and peripheral nervous system. It is characterised by impaired consciousness, ataxia, and ophthalmoplegia [1, 2]. Fisher syndrome (FS) is also an immune-mediated disease characterised by a clinical triad of ophthalmoplegia, ataxia, and areflexia [3]. People with BBE and FS are likely to test positive for serum anti-GQ1b IgG antibodies, an anti-ganglioside antibody, and BBE and FS are potentially on a spectrum with Guillain-Barré Syndrome (GBS). These diseases are essentially sporadic; nevertheless, there are some reports of familial cases of GBS and fewer of FS. However, there are no reports of family cases of BBE and FS.

Here, we report a family case of an 18-year-old son diagnosed with BBE and his 52-year-old mother, diagnosed with FS within 10 days. They were both positive for serum anti-GQ1b IgG antibodies.

## Case presentation

### Case 1: son

An 18-year-old Japanese man was brought to our hospital with impaired consciousness. He was an obese college student with a body mass index of 35 and lived alone. He had no allergies, no medical history, and no family history of neurological diseases. Seven days before presenting at the hospital, he had a sore throat and a cough, although these improved within a few days. The morning he arrived in hospital, he felt weak, and his mother had taken him to the nearest doctor. He was diagnosed with dehydration and received intravenous fluid replacement. After returning home, he had impaired consciousness, and his mother called an ambulance.

**Fig. 1:**
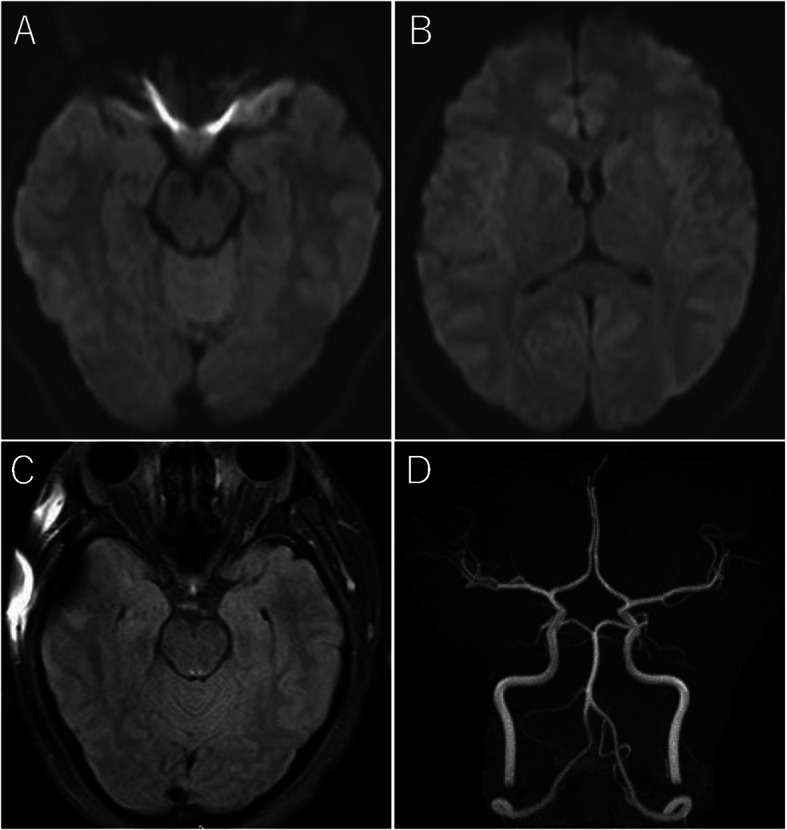
**a**, **b** Case 1: Diffusion-weighted magnetic resonance images: The brain shows no abnormalities. **c**, **d** Case 1: Fluid-attenuated inversion-recovery (FLAIR) images and magnetic resonance angiography (MRA) show no abnormalities

Upon admission, he had a temperature of 37.4 °C and showed a fluctuating state of consciousness, where he sometimes answered simple questions and sometimes went on shouting meaningless words. He presented normal pupils with prompt light reflexes, unrestricted eye movement, no gross muscle weakness including facial muscles, and decreased tendon reflexes in all extremities. Both sides were negative for plantar reflexes. Ataxia could not be assessed because of his fluctuating state of consciousness. There were no signs of meningeal irritation. A laboratory test revealed a C-reactive protein (CRP) level of 1.01 mg/dL (normal range, < 0.5) and a white blood cell (WBC) count of 10.1 × 10^3^ with 71% neutrophils. Cerebrospinal fluid (CSF) analysis revealed a clear appearance, normal opening pressure of 120 mm H_2_O, WBC count of 3 per mm^3^, protein levels of 32 mg/dL, and glucose levels of 62 mg/dL with a CSF/blood glucose ratio of 0.62 (normal range, 0.6–0.8). A non-contrast brain magnetic resonance imaging (MRI) on diffusion-weighted image (DWI) and apparent diffusion coefficient (ADC) revealed no abnormalities. (Fig. 1a, b).

He was started on intravenous acyclovir (3 g/day) due to the possibility of herpes simplex virus (HSV) encephalitis. On day 2, he went into a deep coma and was put on mechanical ventilation. The involuntary movements around his mouth were mainly evoked by stimuli such as suction, and were thought to be due to the cough reflex, but the possibility of anti-n-methyl-d-aspartate (NMDA) encephalitis was also considered. On day 3, the polymerase chain reaction test for HSV in the CSF was negative. Intravenous administration of methylprednisolone (1 g/day) was initiated for 5 days due to the possibility of NMDA receptor encephalitis; however, there was no improvement in his symptoms. On day 7, the CSF anti-NMDA receptor antibodies and the serum anti-AMPH, CV2, PNMA2, Ri, Yo, Hu, recoverin, SOX1, titin, zic4, GAD65 and Tr antibodies were confirmed to be negative. On day 8, a second non-contrast MRI of the brain was taken, which showed no obvious abnormalities on DWI, T1-weighted image (T1-WI), T2-weighted image (T2-WI), fluid attenuated inversion recovery (FLAIR), and T2* and MR angiography (MRA). (Fig. 1c, d). On day 9, a second CSF analysis revealed albuminocytologic dissociation, with elevated protein levels of 111 mg/dL and a normal WBC count of less than 1 per mm^3^, and nerve conduction studies (NCS) revealed motor and sensory axonopathy. We considered BBE and started intravenous immunoglobulin (IVIg) therapy (0.4 g/kg/day) for 5 days. On day 13, he was able to follow orders; however, external ophthalmoplegia, severe muscle weakness of the face and limbs, and ataxia were evident. Serum anti-GQ1b IgG antibodies and anti-GT1a IgG antibodies, tested using an Enzyme-Linked ImmunoSorbent *Assay* (ELISA), were positive on day 15, with optical density (O.D.) value of over 0.459 and 0.235, respectively (normal range, < 0.1). *Haemophilus influenzae (H. influenzae)* was detected on a sputum culture on day 17. We confirmed the diagnosis as BBE and observed the effects of IVIg. His symptoms gradually improved, and he was weaned off the ventilator on day 29. He also began walking exercises later on and was transferred to a rehabilitation hospital on day 65.

### Case 2: mother

A 52-year-old Japanese woman, the mother of Case 1, presented at our department 10 days after her son’s admission. Until then, she was healthy except for a history of hives and a chronic cough. Seven days before the visit, she had a sore throat and a cough. Two days before the visit, she had diplopia; which worsened, so she came to the hospital.

**Fig. 2:**
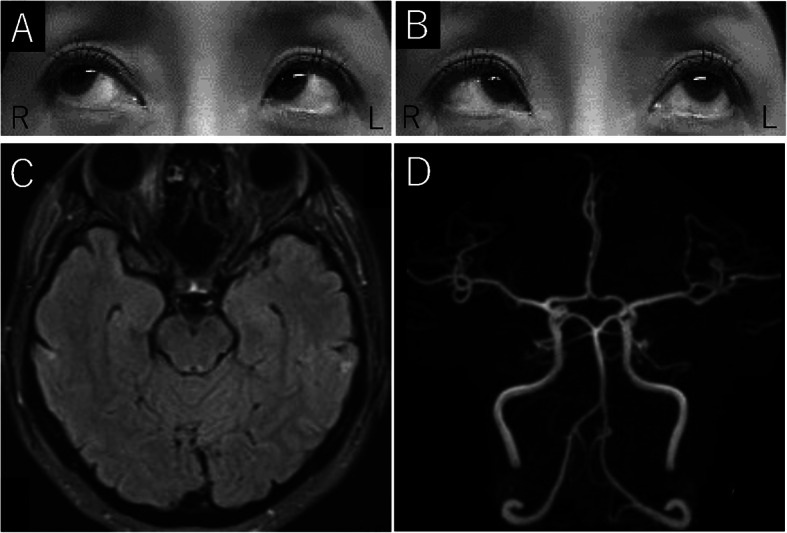
**a**, **b** Case 2: Limited abduction of the left eye. **c**, **d** Case 2: FLAIR images and MRA show no abnormalities

She showed bilateral pupillary dilatation and loss of the light reflex. The abduction of the left eye was limited, and the diplopia worsened when she looked to her left (Fig. 2a, b). Mild ataxia of her left limbs was observed. Her left Achilles’ tendon reflex was decreased. Laboratory tests revealed no elevation of a CRP level and a WBC count. Anti-acetylcholine receptor antibodies and anti-thyroid stimulating hormone receptor antibodies were negative. CSF analysis revealed a clear appearance, normal opening pressure of 10.5 mm H_2_O, WBC count of 4 per mm^3^, protein levels of 26 mg/dL, and glucose levels of 60 mg/dL with a CSF/blood glucose ratio of 0.59. We considered it less likely that myasthenia gravis or Graves’ disease could be the cause of diplopia. NCS revealed no motor and sensory axonopathy or demyelination, which shows that there was no evidence of GBS-like peripheral neuropathy. Brain MRA did not show cerebral aneurysm and brain MRI revealed no abnormal findings on DWI, ADC, FLAIR, T2WI and T2*. (Fig. 2c, d).

We considered a possible diagnosis of FS. We considered the possibility of a mother-son case of FS and BBE, respectively, and restricted family visits (from the mother’s side) to prevent further transmission. Although her symptoms were mild, considering her son’s condition, IVIg therapy (0.4 g/kg/day) for 5 days was initiated on day 2, and her symptoms gradually improved. On day 12, the serum anti-GQ1b IgG antibodies and anti-GT1a IgG antibodies, tested using the ELISA method, were positive, with O.D. value of 0.264 and 1.194, respectively. These findings led to a diagnosis of FS. Although limited abduction of the left eye and decreased left Achilles’ tendon reflex remained, her diplopia, ataxia of the left limbs, bilateral pupillary dilatation, and loss of the light reflex improved after IVIg therapy, and She was discharged on day 14. The human leukocyte antigens (HLA) common to the son and mother were A24, B37, and DR10. Six months after being discharged, the mother’s symptoms have completely disappeared, and the son is walking to school, although he has difficulty running. The other family members, including the son’s father and elder sister, both living apart, had no history of GBS, FS, and BBE.

## Discussion and conclusions

This mother-son case highlights three considerations, as follows: The validity of their diagnosis as FS and BBE, respectively; the link between the son’s BBE and mother’s FS; and host factors and management of contagious risk.

The diagnosis of BBE is made by the presence of all three rapidly-appearing clinical signs (i.e., ataxia, bilateral exophthalmia, and impaired consciousness) and serological anti-GQ1b IgG antibodies with the exclusion of other diseases [4]. In the son’s case, he met the diagnostic criteria for BBE because he had the three signs after a rapid onset of delirium and the detection of anti-GQ1b IgG antibodies. Although delirium as BBE’s initial symptom is uncommon, similar cases have been reported [5, 6]. Haematological and imaging tests were used to exclude the other diseases, and the BBE diagnosis was considered consistent.

The diagnosis of FS is made by the clinical presentation of bilateral ophthalmoplegia, ataxia, and loss of tendon reflexes with the exclusion of other diseases [4], and serological detection of anti-GQ1b IgG antibodies is important as an adjunct for diagnosis. The mother met the clinical diagnostic criteria for FS, and testing positive for serum anti-GQ1b IgG antibodies supported the FS diagnosis.

Next, we consider the relationship between the son’s BBE and mother’s FS. Some familial cases of GBS have been reported [7, 8]. For FS, there is a report of sibling cases (a 2-year-old boy and a 4-year-old girl) diagnosed simultaneously, although anti-GQ1b IgG antibodies were inconclusive [9]. GBS, BBE, and FS have been suggested to be in the same spectrum of disorders related to anti-ganglioside antibodies. In particular, BBE and FS are known to be highly positive for serum anti-GQ1b IgG antibodies (66–68% vs. 83–89%) [1–3, 10]. Anti-GQ1b antibodies are known to be associated with *H. influenzae* infection. In a review of 70 clinically diagnosed cases of FS, all confirmed cases of *H. influenzae* infection were positive for anti-GQ1b IgG antibodies [11]. *H. influenzae* have GQ1b mimics, including a disialosyl group in the non-reducing end of oligosaccharide chain common to GQ1b, in the lipo-oligosaccharide of the cell wall’s outer membrane. This mimicry suggests that molecular homology may be involved in the development of the disease [12, 13]. In our mother-son case, both patients had upper respiratory symptoms as the prodromal symptoms. The son’s sputum culture contained *H. influenzae*. The mother had no sputum production and was unable to provide a specimen for culture. However, after bringing her son to the hospital, she presented with the same upper respiratory symptoms as her son, which indicated that the same organism caused the infection. Both patients had serum anti-GQ1b and anti-GT1a antibodies, which suggested that this was a familial case of BBE and FS due to the *H. influenzae* infection.

When the possibility of FS emerged in the mother, we considered the patients’ genetic factors and the potential risk of transmission and limited family visits, especially for the mother’s side of the family. In sporadic and familial GBS, the influence of the patient’s genetic factors, such as HLA, has been speculated to play a role. Among other autoimmune diseases, it has been reported that MS, type I diabetes, Graves’ disease, discoid lupus, and systemic lupus erythematosus have strong familial associations and they may reflect a common etiologic pathway with shared genetic or environmental influences, including HLA [14]. HLA plays an important role in the immune response by presenting antigens to the T cells, and contributing to T cell differentiation and subsequent antibody production. The diversity of HLA is linked to differences in immune responses to specific antigens, and may be involved in immune responses-related diseases. The fact that the mother and child share some of the same HLA may have led to a similar immune response. There are reports of higher rates of HLA-DR3 and DR7 in sporadic GBS compared to healthy individuals [15, 16]. Otherwise, a report suggests no association between HLA and IgG anti-GQ1b antibody, so the relationship between GBS or its disease spectrum is still controversial [17]. HLA has also been studied in a small number of familial GBS cases; however, no definitive information is available [8]. We examined the patients’ HLA, and A24, B37, and DR10 were commonly identified. In autoimmune diseases, it was reported that A24 was related to autoimmune thyroiditis and DR10 rheumatoid arthritis, but the results were not consistent with previous GBS reports. Although the host factors could not be determined in our cases, it was considered necessary to be aware of inter-family transmission in patients with BBE and FS.

To our knowledge, this is the first report of a mother-son case of FS and BBE. It was suggested that this was a familial case of BBE and FS due to infection with the same organism, *H. influenzae*, and caution should be exercised in BBE and FS cases to avoid inter-family transmission.

## Data Availability

Not applicable.

## Abbreviations

ADC: Apparent diffusion coefficient
BBE: Bickerstaff’s brainstem encephalitis
CRP: C-reactive protein
CSF: Cerebrospinal fluid
DWI: Diffusion-weighted image
ELISA: Enzyme-Linked ImmunoSorbent Assay
FS: Fisher syndrome
GBS: Guillain-Barré Syndrome
*H. influenzae*: *Haemophilus influenzae*
HLA: Human leukocyte antigens
HSV: Herpes simplex virus
IVIg: Intravenous immunoglobulin
MRA: Magnetic resonance angiography
MRI: Magnetic resonance imaging
NCS: Nerve conduction studies
NMDA: N-methyl-d-aspartate
O.D.: Optical density
T1-WI: T1 weighted image
T2-WI: T2 weighted image
WBC: White blood cell
